# A Fatal Case of Louping-ill in a Dog: Immunolocalization and Full Genome Sequencing of the Virus

**DOI:** 10.1016/j.jcpa.2018.09.004

**Published:** 2018-11

**Authors:** M.P. Dagleish, J.J. Clark, C. Robson, M. Tucker, R.J. Orton, M.S. Rocchi

**Affiliations:** ∗ Moredun Research Institute, Pentlands Science Park, Bush Loan, Penicuik, Scotland, UK; † MRC University of Glasgow Centre for Virus Research, Glasgow, Scotland, UK; ‡ Castle Veterinary Group Ltd., Pennygillam Way, Launceston, Scotland, UK

**Keywords:** dog, genome, immunohistochemistry, louping-ill

## Abstract

Louping-ill (LI), caused by louping-ill virus (LIV), results in a frequently fatal encephalitis primarily affecting sheep and red grouse (*Lagopus lagopus scotica*), but it does occur in other species. An adult male Border collie dog was definitively diagnosed with fatal LI and the lesion profile, LIV antigen distribution and full genome sequence of the LIV responsible were investigated to determine if this differed significantly from sheep-derived LIV. No gross lesions were present. The histological lesions were confined to the central nervous system and comprised of lymphocytic perivascular cuffs, glial foci, neuronal necrosis and neuronophagia. Immunolocalization of viral antigen showed small amounts present in neurons only. These histological and immunohistochemical findings were similar to those reported in affected sheep. Compared with published full genome sequences of sheep-derived LIV, only very minor differences were present and phylogenetically the virus clustered individually between a subclade containing Scottish strains, LIV 369/T2 and G and another subclade containing an English isolate LIV A. The LIV isolated from the dog shares a common progenitor with LIV A. These findings suggest there is no canine-specific LIV strain, dogs are susceptible to sheep-associated strains of LI and with the increase in tick prevalence, and therefore exposure to LIV, a safe, effective vaccine for dogs may be required.

## Introduction

Louping-ill (LI) is a frequently fatal encephalitis primarily affecting sheep and red grouse (*Lagopus lagopus scotica*). The causative agent is louping-ill virus (LIV), a single stranded RNA virus of the genus *Flavivirus*, family Flaviviridae, which is transmitted by the sheep tick (*Ixodes ricinus*) (Reid and Chianini, 2007). LI is found predominantly in upland areas of the UK, but is not restricted to this habitat (Jeffries *et al.*, 2014) and has been reported in Norway (Gao *et al.*, 1993a), Spain (Balseiro *et al.*, 2012), the Danish island of Bornholm (Jensen *et al.*, 2004) and even Far-Eastern Russia (Leonova *et al.*, 2015). Closely related viruses have been found also in Spain (Gonzalez et al., 1987, Mansfield et al., 2015), Greece (Pavlidou *et al.*, 2008) and Turkey (Gao *et al.*, 1993b).

LI occurs in non-ovine ruminant species at a much lower incidence, including in cattle (Benavides *et al.*, 2011), goats (Gray *et al.*, 1988), roe (*Capreolus capreolus*) (Reid *et al.*, 1976) and red deer (*Cervus elaphus*) (Reid *et al.*, 1978), alpacas (*Vicugna pacos*) (Cranwell *et al.*, 2008) and llamas (*Lama glama*) (MacAldowie *et al.*, 2005). It occurs rarely in non-ruminant mammals including pigs (Bannatyne *et al.*, 1980), generally inducing non-fatal cases in horses (Timoney *et al.*, 1976), hares (*Lepus timidus*) (Smith *et al.*, 1964) and man (Reid *et al.*, 1972). In dogs, there is a single report of one presumed and one confirmed case of fatal LI in post-parturient working collie dogs (MacKenzie *et al.*, 1973) as well as a case in which the dog recovered (MacKenzie, 1982). The fatal canine case was diagnosed by mouse inoculation of post-mortem harvested brainstem in tissue culture and confirmed by inhibition of virus-induced plaque formation by convalescent sheep serum (MacKenzie *et al.*, 1973). Histological examination was restricted to histochemical-stained sections of the brain as immunohistochemistry (IHC) for LIV was unavailable at that time. In addition, the development of polymerase chain reaction (PCR) and genome sequencing post date this piece of research, so the properties of this LIV isolate and the possibility of different strains of LIV being responsible for LI in dogs and other atypical species could not be examined. Therefore, we took the opportunity to conduct a more in-depth analysis when presented with a new fatal case of canine LI. The aims of this study were to determine: (1) the morphology and distribution of histological lesions in the canine brain, (2) whether IHC used routinely in sheep samples is suitable for definitive diagnosis of LI in dogs and can reveal the distribution of LIV antigen, and (3) whether the LIV responsible for this fatal canine case differed significantly at the genomic level compared with LIV recovered from clinically affected sheep.

## Materials and Methods

### Case History

A male Border collie dog (3 years and 7 months old, body weight 18.8 kg) was observed by the owner to be ‘off-colour’ and ataxic in June 2015. It was presented the following day and was profoundly depressed, ataxic and had an elevated respiratory rate. The animal was hospitalised, given intravenous fluids (1 litre of Hartman's solution) and 9.3 mg/kg amoxicillin and clavulanic acid (Synulox RTU, Zoetis UK Ltd., London, UK) subcutaneously. Blood was taken for serology as LI was suspected due to the dog living and working on a moorland sheep farm located in a LI endemic area. Urine analysis (Multistix 8Sg™; Siemens Healthcare Diagnostics Inc., Tarrytown, New York, USA) and left lateral thoracic radiographs were within normal limits. Despite initial clinical improvement the dog died 19 h after presentation.

### Serology and Molecular Detection of Louping-ill Virus and *Anaplasma phagocytophilum*

Serology for LIV was performed by the haemagglutinin-inhibition (HI) test (Reid and Doherty, 1971a) evaluating both total immunoglobulin (Ig) and IgG fractions by heat-treating a separate aliquot of the serum to dissociate the IgM fraction. Any titre apparent from heat-treated sera is presumed to be due to IgG. Molecular detection of LIV was by subjecting the fresh-frozen samples of brain and spinal cord (see below) to specific TaqMan reverse transcriptase (RT)-PCR (Marriot *et al.*, 2006). A cycle threshold value (Ct) of 35 or less is considered positive for this assay. Molecular detection of *Anaplasma phagocytophilum* was by subjecting DNA extracted from formalin-fixed and paraffin-wax embedded lung, liver and spleen (RecoverAll™ Total Nucleic Acid Isolation Kit for FFPE, ThermoFisher, Waltham, Massachusetts, USA) to specific TaqMan RT-PCR (Courtney *et al.*, 2004).

### Pathology and Immunohistochemistry

Post-mortem examination was performed within 7 h of death and tissue samples, including whole brain, were placed into 10% neutral buffered formalin. Additionally, samples of fresh-frozen frontal lobe and proximal cervical spinal cord were stored at −80°C until required. Coronal slices of fixed brain were made through the anterior pole of the cerebrum, corpus striatum, thalamus, occipital lobes, midbrain, cerebellar peduncles and three levels of the medulla oblongata, plus a sagittal section through the cerebellar vermis. Fixed samples of lung, heart, liver, pancreas, spleen and kidney were also trimmed. All samples were processed routinely and embedded in paraffin-wax. Sections (5 μm) were stained with haematoxylin and eosin (HE). Semiserial sections of all processed tissues were subjected to IHC specific for LIV using a mouse monoclonal antibody (Dagleish *et al.*, 2010). Positive control sections for IHC were from a LIV-positive sheep brain definitively diagnosed by histology, IHC and LIV-specific real-time PCR (https://www.ncbi.nlm.nih.gov/pubmed/16814876). Negative control preparations were semiserial sections of all processed tissues and one of the positive control sample with the primary anti-LIV antibody substituted with normal mouse IgG at the same concentration.

### Genome Sequencing of Louping-ill Virus

Viral RNA was extracted from the fresh-frozen frontal lobe of the brain and spinal cord tissue samples separately using TRIzol™ (ThermoFisher) as per the manufacturer's instructions. Three micrograms of total RNA from each sample were subjected to next generation sequencing (NGS) by initially depleting ribosomal RNA (RiboZeroGold™ kit, Illumina, San Diego, California, USA, according to the manufacturer's instructions) then reverse transcribed using Superscript III Reverse Transcriptase™ (Invitrogen, ThermoFisher). cDNA libraries were generated using the TruSeq Stranded Total RNA Library Prep Kit™ (Illumina) and a modified protocol omitting ribosomal RNA depletion was utilized. This is due to ribosomal RNA depletion relying on the presence of a polyA tail, which LIV lacks. Resultant libraries were quantified using a Qubit 3.0™ fluorometer (Invitrogen, ThermoFisher) and size range determined (2200 TapeStation™, Agilent Technologies LDA UK, Stockport, UK). Libraries, pooled in equimolar concentrations, were sequenced on an Ilumina MiSeq™ (150 base pair [bp] paired end reads).

Prior to bioinformatic analysis, reads were assessed for quality using FASTQC (http://www.bioinformatics.babraham.ac.uk/projects/fastqc/), adapter sequences removed and quality filtered using trim_galore (https://www.bioinformatics.babraham.ac.uk/projects/trim_galore/) with a quality threshold of Q25 and a minimum read length of 75; reads were also filtered for low complexity (reads dominated by a short repeat sequence or an individual nucleotide) duplicates using PRINSEQ (Schmieder and Edwards, 2011). Filtered reads were subsequently mapped onto a LIV complete genome sequence in GenBank (LIV 369, accession number Y07863) using alignment software (BWA, Li and Durbin, 2009). The assembled data were parsed using DiversiTools (http://josephhughes.github.io/btctools/) to determine the frequency of nucleotides at each site and to reconstruct a consensus sequence of the virus. The consensus sequence is defined as the most dominant nucleotide at each genome position, with ambiguity codes only used if two (or more) nucleotides are observed equally at a genome position and N's used at genome positions that had no read coverage. The genome was then extended at the 5′ and 3′ untranslated regions (UTRs) by extracting additional reads that overlapped with the terminal ends of the consensus sequence previously generated. For the brain sample 4,502,776 raw reads (2,251,388 pairs) were obtained, of which 547 mapped to the LIV genome. For the spinal cord sample 5,503,230 raw reads (2,751,615 pairs) were obtained and 257 mapped to the LIV genome.

### Rapid Amplification of cDNA Ends (RACE) Analysis of 5′ and 3′ Genomic Termini and Additional Sequencing

Whole genome sequencing resulted in almost complete coverage of the genome, except for the 5′ and 3′ termini and one region of around 1 kb located within the NS2A gene. To resolve this, primers LIV P7 and LIV P8 were designed to amplify this region, which was then sequenced via commercially available Sanger sequencing (Source Bioscience, Nottingham, UK). In addition, sequencing of the genomic termini was performed using a 5′/3′ RACE PCR kit (Roche, Basel, Switzerland) as per the manufacturer's instructions. For the 5′ RACE, LIV specific primers SP1 and SP2 were utilized (Table 1). As the 3′ RACE relies on the presence of a polyA tail at the 3′ end of the viral genome, one was added using an *Escherichia coli* Poly (A) Polymerase kit (New England Biolabs, Ipswich, Massachusetts, USA) as per the manufacturer's instructions. For the 3′ RACE, LIV specific primer SP5 was used (Table 1). The purified 5′ and 3′ RACE PCR products were then sequenced (Source Bioscience).

**Table 1:** Primers used in this study

Primer name	Sequence (5′-3′)	Use
SP1 (R)	CCCATCATGCGCATCAATA	5′ RACE
SP2 (R)	GCCCCCCTTGCCTTTCAGGA	5′ RACE
SP5 (F)	GGGAGCTCAAGCTAGAGAGC	3′ RACE
P7 (F)	CACAATAAATGCCAAGTGTGAAAA	Sequencing gaps
P8 (R)	AACCAGCTGCATCTTCCTCG	Sequencing gaps
Seq 1 (F)	GTTGTGCTCCTGTGTTTGGC	Diagnosis
Seq 2 (R)	CCACTCTTCAGGTGATACTTGTTTCC	Diagnosis

F, forward primer; R, reverse primer.

### Phylogenetic and Sequence Analysis

Phylogenetic and sequence analyses were performed using full coding sequence alignments generated using MUSCLE (Edgar, 2004) within the program suite Geneious (version 7.1.8: http://www.geneious.com; Kearse *et al.*, 2012). The brain and spinal cord samples were each aligned to the LIV reference sequence 369 (accession number Y07863). The sequences produced from the 5′ and 3′ RACE analysis and the internal sequencing PCRs were then aligned to these and consensus sequences were generated. For the phylogenetic analysis, a MUSCLE alignment was generated using the dog brain sample in addition to all other publicly available LIV sequences in Genbank (https://www.ncbi.nlm.nih.gov/genbank/). All sequence names and accession numbers are shown in Table 2. Spanish sheep encephalitis virus (SSEV, accession number DQ235152) was included as an outgroup. This alignment was analysed for the presence of recombination using the Recombination Detection program 4 (RDP4) software package (Martin *et al.*, 2015), specifically the programs RDP, Chimeara, BootScan, 3Seq, GENECOV, MacChi and SiScan were used. A maximum likelihood tree was generated using the programs PhML within the Geneious software package (Guindon and Gascuel, 2003). Support for the maximum likelihood tree topology was generated by 1,000 non-parametric bootstrap replicates. The generalized time reversible (GTR) substitution model with gamma distribution (+G) was found to suit the dataset best, as selected by both jModel test (Darriba *et al.*, 2012) and HyPhy software packages (Pond *et al.*, 2005).

**Table 2:** Flavivirus full genome and ENV sequences utilized in this study

Sequence name	Sequence description	Accession number
LIV Dog	Full genome sequence	MH537791
LIV 3/1	Full genome sequence	KP144331
LIV 369 (T2)	Full genome sequence	Y07863
LIV LEIV-7435Tur	Full genome sequence	KT224354
LIV Penrith	Full genome sequence	KF056331
LIV Primorye-185-91	Full genome sequence	KJ495985
Negishi	Full genome sequence	KT224355
SGEV	Full genome sequence	KP144332
SSEV	Full genome sequence	DQ235152
GGEV	Full genome sequence	DQ235153
TBEV Neudoerfl	Full genome sequence	U27495
TBEV Hypr	Full genome sequence	KP716978
TBEV Tobrman	Full genome sequence	KJ922515
TBEV Absettarov	Full genome sequence	KJ000002
TBEV KrM 93	Full genome sequence	HM535611
TBEV KrM 213	Full genome sequence	HM535610
TBEV Vlasaty	Full genome sequence	KJ922516
TBEV Sofjin	Full genome sequence	JF819648
TBEV Senzhang	Full genome sequence	JQ650523
TBEV Xinjiang	Full genome sequence	JX534167
TBEV Sib-XJ-X5	Full genome sequence	KP345889
OHFV Bogoluvovska	Full genome sequence	NC_005062
LIV 31	ENV gene sequence	D12937
LIV 261	ENV gene sequence	X86787
LIV 917	ENV gene sequence	X86786
LIV G	ENV gene sequence	X86788
LIV I	ENV gene sequence	X86785
LIV K	ENV gene sequence	D12935
LIV M	ENV gene sequence	X71872
LIV MA54	ENV gene sequence	X86784
LIV A	ENV gene sequence	X69975
LIV NOR	ENV gene sequence	D12936
LIV Primorye-20-79	ENV gene sequence	KJ495984
LIV Primorye-155-77	ENV gene sequence	KJ495983
LIV SB526	ENV gene sequence	M94957

## Results

### Serology and Molecular Identification of Louping-ill Virus

The HI serology test gave a positive titre of 80 for total Ig and absence of IgG, denoting that the immunoglobulin present was IgM and suggesting that the infection was in the acute/subacute stage (IgM predominance). Samples of brain and spinal cord were both positive for LIV RNA by TaqMan RT-PCR, with CT values of 18.35 and 21.97, respectively. Samples of lung, liver and spleen were all negative for *A. phagocytophilum* DNA.

### Pathology and Immunohistochemistry

**Fig. 1:**
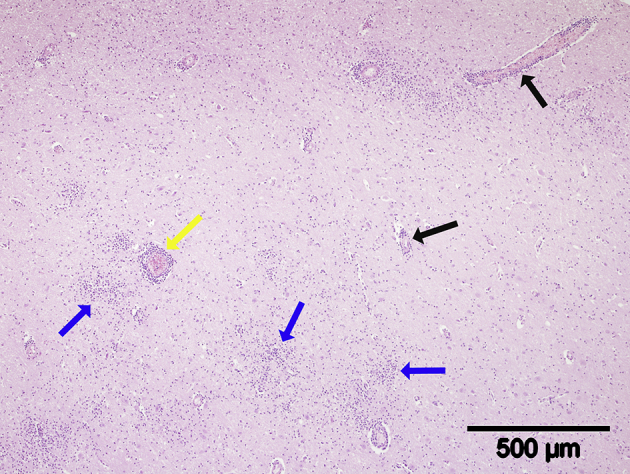
Midbrain. Note thin (black arrows) and medium-sized (yellow arrow) mononuclear cell (presumed lymphocytes), perivascular cuffs and numerous, poorly delineated, glial foci (blue arrows). HE.

**Fig. 2:**
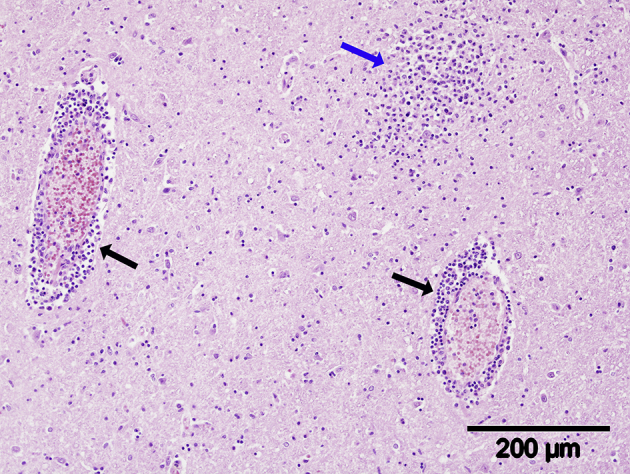
Pons. Note perivascular cuffs comprised of lymphocytes (black arrows) and large glial foci (blue arrow) comprised of mononuclear inflammatory cells (presumed lymphocytes). HE.

No gross lesions were present. Histologically, the brain tissue was mildly autolytic and contained a very large number of thin to medium, occasionally thick, lymphocytic perivascular cuffs throughout the cerebrum and brainstem, which were more numerous in the grey matter than the white (Fig. 1) and thickest in the midbrain and pons regions. Many variably sized, but mainly large, mononuclear cell glial foci with indistinct borders were present in the grey matter throughout the brain (Fig. 2) and small numbers of necrotic neurons with neuronophagia were also present within glial foci. The cerebellum was minimally affected by these lesions, but a small number of variably sized, small to large haemorrhages were present in the internal granular and molecular layers of the caudoventral cerebellar vermis and the medulla. Severe, generalized congestion was present in the lung, liver and kidney and mild to moderate congestion in the pancreas, which contained a small number of randomly distributed small haemorrhages. The red pulp of the spleen was severely depleted and a very small number of small to medium-sized periarteriolar lymphoid sheaths was present. No significant lesions were present in the section of heart examined. A morphological diagnosis of severe, acute to subacute, generalized, lymphocytic panencephalitis was made.

**Fig. 3:**
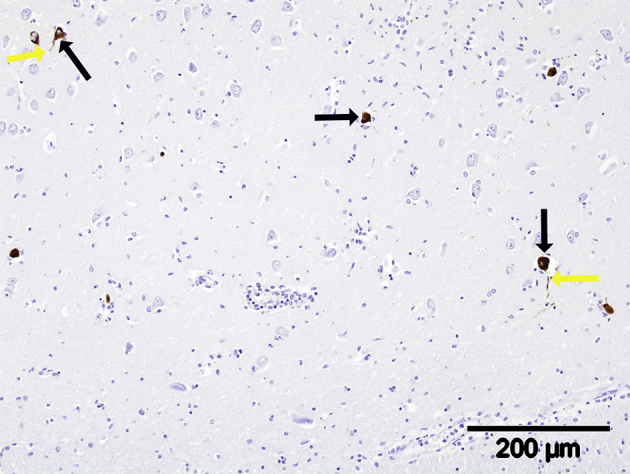
Specific immunohistochemistry for LIV antigen. Hippocampus. Note the discrete labelling of the cytoplasm of neurons (black arrows), which can be seen extending into their associated axons (yellow arrows).

Despite the severe and extensive histological lesions, IHC for LIV antigen showed only small amounts of positive labelling, which were confined to the cytoplasm of neurons and their axons, most frequently in the hippocampus (Fig. 3) and cerebellar peduncles, with occasional Purkinje cells in the cerebellum also being positive. All visceral tissues and negative control preparations were devoid of immunolabelling.

### Genome Sequencing and Comparison with Sheep-derived Louping-ill Virus

**Fig. 4:**
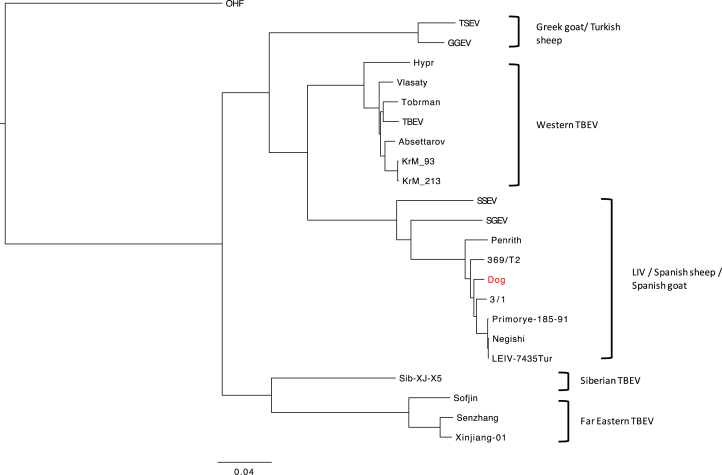
Phylogenetic tree generated using tick-borne flavivirus full genome sequences. The maximum likelihood tree was generated using PHYML within the software suite Geneious. Branch support values are denoted utilizing 1,000 bootstrap replicates; bootstrap values 50% are displayed on branches. LIV Dog is highlighted in red. Omsk haemorrhagic fever virus (OHF) was included as an outgroup.

**Fig. 5:**
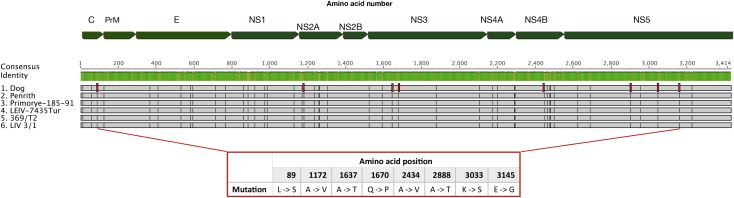
Pairwise alignment of full LIV polypeptide sequences with LIV Dog. The mean pairwise identity of all amino acids at a given position is indicated by the identity bar; green corresponds to 100% pairwise identity, yellow highlights positions possessing <100% pairwise identity. Pairwise sequence identity is denoted by black bars (>60% similarity) and grey bars (60–80% similarity). The position of the eight amino acid substitutions unique to LIV Dog are highlighted in red. Accession numbers corresponding to the strains included in this analysis are shown in Table 2.

**Fig. 6:**
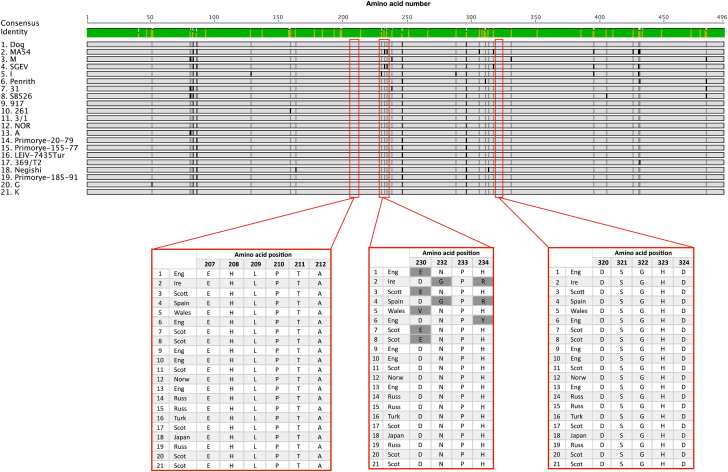
Pairwise alignment of LIV ENV amino acid sequences with LIV Dog. The mean pairwise identity of all amino acids at a given position is indicated by the identity bar; green corresponds to 100% pairwise identity, yellow highlights positions possessing <100% pairwise identity. Pairwise sequence identity is denoted by black bars (>60% similarity) and grey bars (60–80% similarity). The tick-borne flavivirus-specific hexapeptide (amino acids 207–2,012) and pentapeptide (amino acids 320–324) sequences are highlighted, in addition to the LIV-specific tripeptide motif, (amino acids 230–234) and the region-specific amino acid at position 230. Each strain is numbered 1–21 and the geographical location of the strain is detailed as: Eng (England), Ire (Ireland), Scott (Scotland), Spain, Wales, Norw (Norway), Russ (Russia), Turk (Turkmenistan) and Japan. Accession numbers corresponding to the strains included in this analysis are shown in Table 2.

**Fig. 7:**
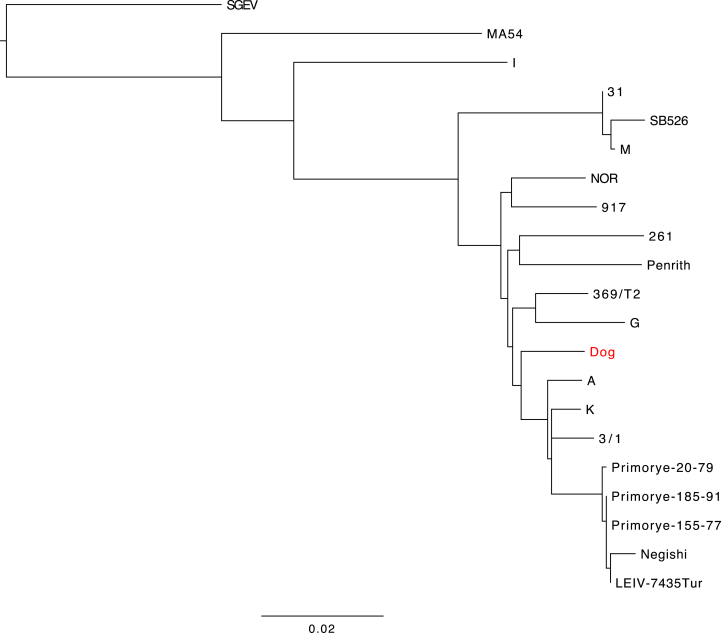
Phylogenetic tree generated using LIV ENV sequences. Note LIV Dog (highlighted in red) is clustered individually between a subclade containing Scottish strains, LIV 369/T2 and G and another subclade containing English isolate LIV A, and that LIV Dog shares a common progenitor with LIV A. Maximum likelihood tree generated using PHYML within the software suite Geneious. Branch support values are denoted utilizing 1,000 bootstrap replicates; bootstrap values over 50% are displayed on branches. Spanish goat encephalitis virus (SGEV) was included as an outgroup.

Full genome sequencing followed by phylogenetic analysis showed that the LIV isolate from the dog (denoted LIV DOG, accession number: MH537791) shared a 98.1% identity with LIV Scottish isolate 369/T2 (accession number: NC_001809) and clustered between this and English isolate 3/1 (Fig. 4). Full genome comparison via multiple sequence alignment with LIV genomes 3/1, 369/T2, Penrith, Primorye-185-91 and LEIV-7435 (accession numbers in Table 2) uncovered eight non-synonymous mutations scattered throughout the genome, which were unique to LIV DOG (Fig. 5). Most of these changes resulted in the substitution of a neutrally charged amino acid by another neutrally charged amino acid, except for residue 3033 located within the NS5 gene, which resulted in the substitution of a neutrally charged lysine in place of a positively charged serine. At the nucleotide level, LIV DOG shared higher homology with isolates LIV 3/1, Primorye-185-91 and LEIV-7435 compared with LIV 369/T2 and LIV Penrith, than isolate 369/T2 (Table 3). Pairwise alignment of multiple LIV ENV gene sequences (accession numbers in Table 2) revealed that the tick-borne flavivirus-specific peptide motifs EHLPTA (amino acids 207–212 in the LIV E gene) and DSGHD (amino acids 320–324 in the LIV E gene) were conserved in the LIV DOG ENV sequence, as was the LIV-specific tripeptide NPH (amino acids 232–234 in the LIV E gene) (Fig. 6) (Shiu et al., 1991, Shiu et al., 1992, Venugopal et al., 1992, Gritsun et al., 1993, Gao et al., 1993b, McGuire et al., 1998). The preceding region-specific peptide (amino acid 230) is glutamine (E), which is associated with Scottish LIV strains (Fig. 6) (McGuire *et al.*, 1998). Phylogenetic analysis of these LIV ENV sequences showed that LIV DOG clustered individually between a subclade containing Scottish strains LIV 369/T2 and G and another subclade containing English isolate LIV A, and that LIV DOG shares a common progenitor with LIV A (Fig. 7), which was isolated in 1980 from a sheep in Devon, which is geographically close to where LIV DOG was found (McGuire *et al.*, 1998).

**Table 3:** Pairwise percentage identity of the full genome sequence of LIV Dog compared with published full length LIV genomes

Isolate	369/T2	Penrith	3/1	Primorye-185-91	LEIV-7435
*% Identity (nt)*					
Dog	96.5	97.0	98.2	98.1	98.2

nt, nucleotide.

## Discussion

This is the first description of the lesion profile, immunolocalization of LIV antigens and the relationship between these in a case of canine LI and the first comparison of the genome of the recovered LIV strain from a dog with those published previously. Additionally, this is only the second report of a definitely diagnosed fatal case of canine LI. That both the present and previously reported definitive and presumptive (MacKenzie *et al.*, 1973) fatal canine LI cases occurred in working Border collies might suggest a breed predisposition for the disease. However, the level of exposure to LIV-infected sheep ticks is likely much greater in working sheepdogs in the UK compared with the general domestic dog population and probably higher also than other working dogs, such as gun dogs, as the latter will not always be exposed to ixodid tick-infected pastures. Physiological stress may play a role in the development of clinical disease, similar to what has been shown in experimental LIV challenge in sheep (Reid and Doherty, 1971a, Reid and Doherty, 1971b), as the fatal canine cases previously reported were both in post-parturient bitches (MacKenzie *et al.*, 1973). However, that does not explain the present case, which was a young adult male with no known previous or intercurrent health problems. Coinfection with *A. phagocytophilum* has been shown to exacerbate the lesions of LI in sheep, which was presumed due to immunosuppression (Reid *et al.*, 1986), but there was no evidence of this in the present case. The relative paucity of clinical signs in the present case is typical of animals that succumb in the acute phase of the disease, 5–7 days post infection (Doherty and Reid, 1971a, Doherty and Reid, 1971b).

The morphology, number and distribution of the brain lesions are consistent with those found in sheep with LI (Doherty and Reid, 1971a, Doherty and Reid, 1971b) and also in several other species (Macaldowie et al., 2005, Benavides et al., 2011). Additionally, the total lack of immunolabelling of LIV in any tissue except the central nervous system, together with the limited immunolabelling within the brain, is not inconsistent with ovine clinical field cases (M. Dagleish, personal observations) and previous experimental studies in sheep, as large amounts of LIV antigen are usually found by IHC only in the peracute stage of disease (Reid and Doherty, 1971a). Once moderate lymphocytic lesions have developed in the brain, immunolabelling of LIV has been shown to be sparse, due to the production of IgM and IgG, which quenches the virus (Reid and Doherty, 1971a, Doherty and Reid, 1971b).

The exceptionally high full genome nucleotide similarity of LIV DOG with the other sheep- and tick-derived published genomes of LIV (96.5–98.2, Table 3), its phylogenetic location within them rather than as an outlier (Fig. 4), the sharing of a common progenitor with an LIV strain isolated from a sheep in close geographical proximity and the presence of the tick-borne flavivirus-specific peptide motifs (Fig. 6, McGuire *et al.*,1998), all suggest that it is highly unlikely that LIV DOG is a canine-specific strain or specifically more highly pathogenic to dogs. Although eight unique amino acid substitutions were identified from the genome in LIV DOG, only one would result in a change of amino acid residue charge; from a neutrally charged lysine to a positively charged serine at residue 3033 in the NS5 gene. The consequences of this are unclear, but it may have resulted in altered protein folding. However, determination of this was beyond the scope of this study. Although the LIV DOG full genome does not differ dramatically from those isolated from sheep, it is interesting to note that it exhibits the LIV-specific peptide marker ENPH, which is associated with Scottish LIV strains. As LIV DOG was isolated in Southern England, but exhibits the Scottish peptide sequence, and as it groups between Scottish and English LIV strains, this may suggest that LIV DOG represents an evolutionary midpoint between Scottish and English LIV strains. The possibility of LIV strains being moved around the UK by sheep trade is discussed elsewhere (McGuire *et al.*, 1998).

In conclusion, the strain of LIV responsible for this fatal case of canine LI was not notably different at the genome level to other strains of LIV isolated from sheep and ticks. Furthermore, the severity, distribution and morphology of the lesions were indistinguishable from those found in sheep, as was the abundance and cellular distribution of LIV antigen by IHC at a similar stage of the disease. This suggests that although large numbers of working sheepdogs may be exposed to LIV, become infected and probably seroconvert, fatal cases are rare, as deaths in such economically valuable animals are likely to be investigated fully. A similar situation occurs in sheep, in that most, if not all, animals in areas endemic for LIV will become infected and seroconvert, which is maintained for life, but only a relatively small number develop fatal disease (Buxton and Reid, 2017). Further studies should include large scale serological testing of working sheepdogs, together with other working and non-working dogs, to determine the incidence of exposure to LIV and to evaluate whether development of a safe, dog-specific vaccine is warranted.
